# The rise of the middle author: Investigating collaboration and division of labor in biomedical research using partial alphabetical authorship

**DOI:** 10.1371/journal.pone.0184601

**Published:** 2017-09-14

**Authors:** Philippe Mongeon, Elise Smith, Bruno Joyal, Vincent Larivière

**Affiliations:** 1 École de bibliothéconomie et des sciences de l’information, Université de Montréal, Montréal, Québec, Canada; 2 Department of Mathematics and Statistics, McGill University, Montréal, Québec, Canada; 3 Observatoire des Sciences et des Technologies (OST), Centre Interuniversitaire de Recherche sur la Science et la Technologie (CIRST), Université du Québec à Montréal, Succ. Centre-Ville, Montréal, Québec, Canada; Johannes Gutenberg Universitat Mainz, GERMANY

## Abstract

Contemporary biomedical research is performed by increasingly large teams. Consequently, an increasingly large number of individuals are being listed as authors in the bylines, which complicates the proper attribution of credit and responsibility to individual authors. Typically, more importance is given to the first and last authors, while it is assumed that the others (the middle authors) have made smaller contributions. However, this may not properly reflect the actual division of labor because some authors other than the first and last may have made major contributions. In practice, research teams may differentiate the main contributors from the rest by using partial alphabetical authorship (i.e., by listing middle authors alphabetically, while maintaining a contribution-based order for more substantial contributions). In this paper, we use partial alphabetical authorship to divide the authors of all biomedical articles in the Web of Science published over the 1980–2015 period in three groups: primary authors, middle authors, and supervisory authors. We operationalize the concept of middle author as those who are listed in alphabetical order in the middle of an authors’ list. Primary and supervisory authors are those listed before and after the alphabetical sequence, respectively. We show that alphabetical ordering of middle authors is frequent in biomedical research, and that the prevalence of this practice is positively correlated with the number of authors in the bylines. We also find that, for articles with 7 or more authors, the average proportion of primary, middle and supervisory authors is independent of the team size, more than half of the authors being middle authors. This suggests that growth in authors lists are not due to an increase in secondary contributions (or middle authors) but, rather, in equivalent increases of all types of roles and contributions (including many primary authors and many supervisory authors). Nevertheless, we show that the relative contribution of alphabetically ordered middle authors to the overall production of knowledge in the biomedical field has greatly increased over the last 35 years.

## Introduction

With the increasing costs, complexity and interdisciplinarity of modern science [1], research collaboration has become the norm [2]. Scientific knowledge is now being produced by increasingly large teams [3,4], often involving researchers from multiple disciplines, institutions and countries [5]. Many funding agencies encourage and facilitate collaboration [6–8] and there is evidence that funded research is indeed more collaborative [9,10]. A growing body of evidence also suggests that collaborative research has more impact and that increasingly large and diverse teams are necessary to achieve greater impact [4].

Larger teams translate into a larger number of authors listed in the byline of scholarly articles. The term ‘team size’ is thus used hereafter to refer to the number of authors on an article. In certain cases, there may be hundreds of authors on a paper; a phenomenon coined as ‘hyperauthorship’ [11]. Larger teams, but also the diversity of collaboration types [12], team composition [13], and work division within the team [14], greatly complicates the attribution of credit and responsibility to individual team members [15]. This is an important issue since the advancement of researchers’ careers largely depends on the credit they obtain for their work [16,17]. Because it is so important, conflicts regarding authorship are becoming commonplace [16,17] and may introduce tensions in the workplace. The growing complexity of credit attribution is also potentially detrimental for the scientific system as a whole, which works best when excellence is properly identified and rewarded [18].

While it may be difficult for an external observer to assess the respective contributions of individual authors of a collaborative work, their position on the byline may be used as a proxy for the extent and nature of their contributions, since names are typically ordered following implicit disciplinary norms [19]. For example, in the biomedical field, as in most lab-based disciplines, authorship order is based on the importance and type of the contribution as well as the hierarchical position within the team or laboratory. Generally, the first and last position are given the most importance. The first author is a PhD student or a postdoctoral fellow who contributed most to the research, and the last is the lab director [20]. Between the first and last authors are listed an increasingly large number of ‘middle authors’ who typically played a less significant role in the research [14]. Another way to obtain information about individual authors’ contributions to a given work is the contribution statement that many scientific journals (e.g., JAMA, BMJ, the Lancet, NEJM and PLoS) require. These statements are intended to provide information about an individual author’s contribution. However, their value is limited because of significant reporting biases [21,22], and because they address the type of work performed by each author but not the relative value or importance of the work. Nonetheless, several analyses of the relation between the authors rank on the byline and their reported contributions [e.g., 14,23] confirmed the polarization of ‘core’ contributors towards the first and the last position of the authors list, while authors who made fewer types of contributions were listed in the middle. Therefore, in this paper we divide the bylines of biomedical articles into three distinct groups using a terminology similar to the one proposed by Baerlocher and colleagues [23]:

1. *Primary authors*: main contributors to the experimental work;
2. *Supervisory authors*: senior researchers who supervised the research; and
3. *Middle authors*: individuals with relatively small contributions to the research who are listed between the primary and supervisory authors.

This raises a difficult question: how can we distinguish primary, middle and supervisory authors? In other words, where does the middle begin and where does it end? Previous bibliometric analyses of biomedical research [24–26] have typically avoided this question by defining the middle authors as all those listed between the first and last position. This poorly reflects reality since it allows only one primary author and only one supervisory author. This is problematic, as collaborative research (especially inter-institutional or interdisciplinary research) is likely to have multiple primary authors leading perhaps different part of the experimental work, and also multiple supervisory authors [27]. Thus, the ‘first author + middle authors + last author’ model is an arbitrary division of authors that might unfairly tag as middle authors some researchers who played major roles in the research.

In this paper, we use partial alphabetical authorship as a tool to identify the primary, middle, and supervisory authors in a given team. As Harriet Zuckerman [28] pointed out, listing a subset of authors in alphabetical order creates a clear distinction between those who are listed alphabetically and those who are not. For instance, if an article has twenty authors, and the six main contributors (the first four and the last two) are not listed in alphabetical order, while authors from the fifth to the eighteenth position are, a distinction is made; the sequence of authors in alphabetical order in the middle of the byline serves to distinguish the primary, middle and supervisory authors. In this paper, the term ‘primary authors’ thus refers to those authors appearing before an alphabetical sequence, ‘middle authors’ refers to those listed in the alphabetical sequence, and ‘supervisory authors’ refers those listed after the alphabetical sequence.

The purpose of this study is to empirically explore the relative contribution of primary authors, middle authors and supervisory authors to research articles in the biomedical field. More specifically we provide answers to the following research questions:

1. How prevalent are alphabetically ordered middle authors in biomedical research?
2. What are the proportions of primary, middle and supervisory authors in the articles’ bylines?
3. How has the overall contribution of middle authors to the biomedical literature evolved over the last 35 years?

## Methods

### Data

This study is based on all biomedical research and clinical medicine articles published between 1980 and 2015, which were authored by 4 to 100 individuals, and indexed in Clarivate Analytics’ Web of Science (WoS). Access to the WoS data in a relational database format was provided by the *Observatoire des sciences et des technologies* (http://www.ost.uqam.ca). The discipline of the articles was determined by the NSF classification of the journal in which they are published. Because trends observed were almost identical in the two biomedical disciplines studied (Biomedical Research and Clinical Medicine), they are combined in the results presented below. We identified middle authors using the following three steps: 1) identifying alphabetical sequences, 2) correcting broken sequences, and 3) distinguishing intentional and incidental alphabetical sequences. While we used proprietary WoS data for this study, other investigations could be performed using non-proprietary data such as PubMed, which also provides an extensive coverage of biomedical literature.

### Identifying middle authors

We used an approach similar to that of Waltman [29] to detect sequences of authors in alphabetical order by giving each author of a byline an alphabetical rank based on their last name, and then their initials. An alphabetically ordered sequence of authors is formed when a group of consecutive authors are listed in alphabetical order. Consider for example, an article authored by Wilson, B., Smith, J., Albert, S., Carter, B., Miller, D., Ford, R., and Clark, P.; it includes a group of three authors (Albert, S., Carter, B., and Miller, D.) in alphabetical order starting from the 3^rd^ position and ending at the 5^th^ position.

### Correcting broken sequences

**Fig 1:**
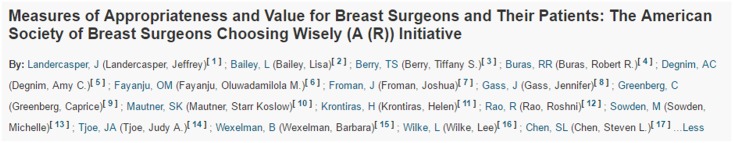
Example of a sequence break due to multiple last names.

Depending solely on names and initials to identify alphabetical sequences has some limitations. Errors can occur because of special character conversion, compound names and names with prefixes, indexation errors, and human errors in the alphabetical ordering. In our dataset, spaces and hyphens are removed from last names (e.g. van Gogh becomes vanGogh), and special characters are converted into the basic Latin alphabet (e.g. Lübeck becomes Luebeck). Also, the prefixes of Dutch names (e.g., van, von) are not taken into account in the alphabetical ordering. It may also happen that the first of two last names of an author is treated as a second first name during the indexation process. Fig 1 shows an example where authors from the second to the second to last positions have been ordered alphabetically. However, the sequence breaks at the 10^th^ author (Starr Koslow Mautner) because her last name (Koslow) has been indexed as a second initial. There may also be cases of human errors, for example when two names are inverted in a long list of otherwise alphabetically ordered authors. Finally, alphabetical ordering conventions differ by language and country, so different individuals may alphabetically order the same list of names in a different way. These conventions also contain rules regarding alphabetical ordering of special characters, which can create further errors since these characters are no longer present in the indexed names.

To reduce as much as possible the occurrence of the errors mentioned above, we concatenated consecutive alphabetically ordered sequences which met one of the following conditions:

$$\left(R=8\;and\;X\leq Y_{1}\leq Z\right)$$

*or*

$$\left(R=6\;and\;X\leq Y_{2}\leq Z\right)$$

*or*

$$\left(R=6\;and\;X\leq Y_{3}\leq Z\right)$$

Where:

- *R* is the combined length (*r*) of the alphabetical sequences preceding and following the break.
- *X* is the first letter of the author name before the one causing the break.
- *Y*_*1*_ is the first letter of the author name causing the break.
- *Y*_*2*_ is the first letter of the author name causing the break after removing potential prefixes.
- *Y*_*3*_ is the last initial of the author name causing the break.
- *Z* is the first letter of the author name after the one causing the break.

The value of *R* is important because the longer the consecutive sequences, the higher the probability that they actually constitute a single sequence that has been broken into two distinct parts. Therefore, to maximize the precision of the alphabetical sequence break detection, we manually verified a random sample of 100 broken alphabetical sequences for different values of *R*, and we selected the minimum value of *R* for which the proportion of false positive was 5% or lower. A total of 192,716 broken alphabetical sequences were fixed: 28,779, 77,332 and 86,605 sequences for which the (*R* = 8 *and X* ≤ *Y*_1_ ≤ *Z*), (*R* = 6 *and X* ≤ *Y*_2_ ≤ *Z*), and (*R* = 6 *and X* ≤ *Y*_3_ ≤ *Z*) conditions were met, respectively. The resulting dataset comprises more than 6.7 million articles authored by a total of more than 44 million authors, among which 13 million alphabetical sequences where found.

### Probability of intentional vs. chance alphabetical order

**Fig 2:**
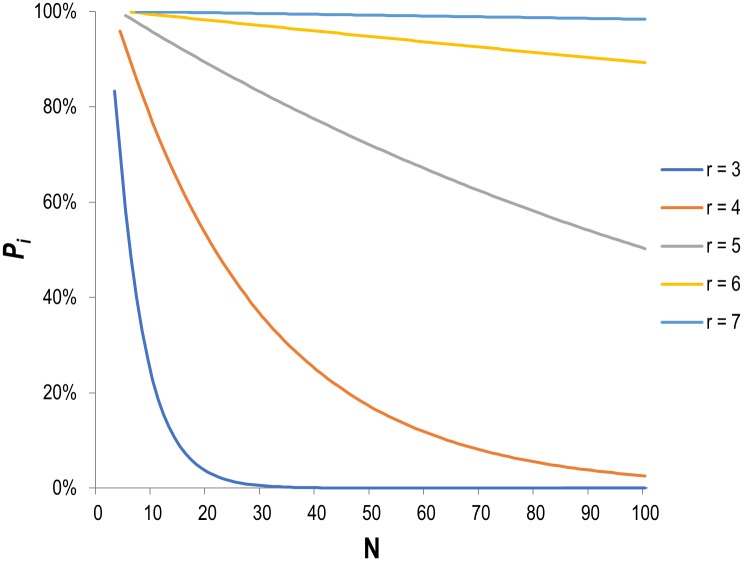
Probability of intentional alphabetical order (*P*_*i*_) as a function of the team size (*N*) and the length of the alphabetical sequence (r).

There is always a possibility that a given sequence of authors in alphabetical order results from pure chance and is not intentional. Distinguishing intentional and chance alphabetical order is crucial since alphabetical sequences that occur randomly cannot be used to distinguish middle authors from the others. Thus, for each sequence found, we calculated *P*_*i*_, which is the probability that the authors are intentionally listed in alphabetical order, and the opposite of the probability *P*_*c*_ that authors are listed alphabetically by chance. *P*_*i*_ is determined by two variables: the number of authors in the sequence (*r*) and the team size (*N*). For example, there are 3,628,800 possible combinations of *N* = 10 authors out of which 156,002 contain an alphabetical sequence of *r* = 5 authors. Thus, a sequence of *r* = 5 has a 156,002/3,628,800 = 4.3% probability of occurring by chance (*P*_*c*_), and therefore a 95.7% chance of being intentional (*P*_*i*_). Fig 2 shows the relation between *N* and *P*_*i*_ for different values of *r*. We see that for short alphabetical sequences of 3 or 4 authors *P*_*i*_ increases rapidly as the byline gets longer, while the *Pi* of sequences of 6 and 7 remains very high, even for articles with up to 100 authors. More details on the calculation of *P*_*i*_ and *P*_*c*_ can be found in the supporting information (S1 File)

The data file resulting from these methodological steps is described in Table 1.

**Table 1:** Description of each field of the data file used for analysis.

Field	Description
Id_article	Unique identifier of the article
*N*	Team size (Total number of authors in the byline)
Pub_year	Publication year of the article
Id_seq	Unique identifier of the alphabetical sequence
Seq_begin	Rank in the byline of the first author of the alphabetical sequence
Seq_end	Rank in the byline of the last auhtor of the alphabetical sequence
*R*	Number of authors in the alphabetical sequence
*P*_*i*_	Probability that the alphabetical sequence is intentional

The identification of primary, middle and supervisory authors through the use of alphabetical subsets does, however, have limitations. One might argue for instance that there are other reasons for this type of ordering. There may be cases where a high number of researchers substantially contributed to different parts of a large project, so that attempting to list authors according to their relative contribution becomes too complex, or a seemingly excessive burden. Researchers may feel that it is simpler—or maybe even fairer—to list some of the authors in alphabetical order. Even if alphabetically ordering middle authors was not initially intended, the result is the same: The primary and supervisory authors are listed in an order that reflects their contribution, and a group of middle authors are listed in alphabetical order. Inversely, middle authors may not be listed in alphabetical order, thus making it impossible to identify them using this method.

## Results

**Fig 3:**
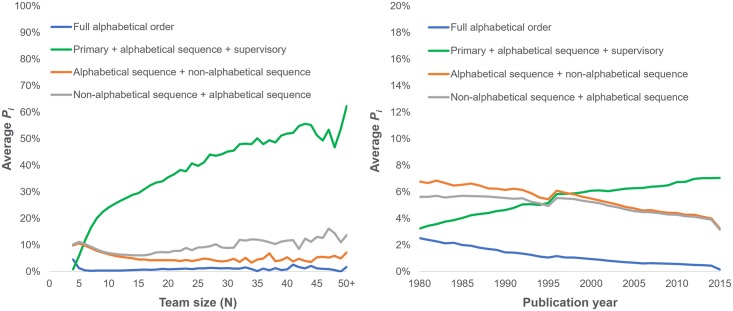
Proportion of article bylines containing intentional alphabetical order as a function of team size (left), and publication year (right).

Fig 3 shows the probability of finding an intentional alphabetical sequence in an article’s byline as a function of the team size (left) and as a function of the publication year (right). We distinguish here cases where middle authors are listed in alphabetical order from cases where the alphabetical sequence begins with the first author or ends with the last authors, as well as cases where all authors are in alphabetical order. Results suggest that alphabetical sequences occur more frequently in the middle of the authors list, and that their prevalence correlates with the team size. The average *P*_*i*_ quickly reaches 25% at *N* = 7 and increases to 50% for *N* = 35. This confirms that it is common practice in the biomedical field to order middle authors in alphabetical order, and more so as team size increases. The other types of alphabetical patterns remain relatively rare.

The right panel of Fig 3 indicates that the proportion of articles with alphabetically ordered middle authors has increased steadily over the last 35 years, rising from approximately 3% of articles in 1980 to almost 8% in 2015. Inversely, the number of bylines in full alphabetical order and of bylines where the first or last authors are in an alphabetical sequence have decreased.

The average size of teams producing biomedical articles varies over time, which may have an effect on the trends observed in Fig 3. To control for this variation, we performed a binomial logistic regression to measure the effect of the team size and the publication year on the probability that the middle authors are ordered alphabetically on a byline with 95% certainty (*P*_*i*_ ≥ 0.95). In order to maintain this level of certainty, the test was performed on the subset of 2,527,997 articles authored by 7 to 100 individuals (the lowest *r* and *N* values for which *P*_*i*_ ≥ .95 are 5 and 7, respectively). The model was statistically significant χ2(2) = 6.61x10e4, p <.001 but explained only 8.6% of the variance in the presence of an alphabetical sequence of authors in the bylines, correctly classifying only 3.4% of cases. As shown in Table 2, the year of publication has in fact no effect on the proportion of bylines with alphabetically ordered middle authors (Exp(B) = 1.001). However, the team size does have an effect (Exp(B) = 1.145) and was the only statistically significant predictor.

**Table 2:** Logistic regression predicting the likelihood of an article containing alphabetically ordered middle authors based on the team size and the publication year.

	B	S.E.	Wald	df	Sig.	Exp(B)	95% C.I.for EXP(B)	
							Lower	Upper
Team size (N)	.135	.001	58258.286	1	.000	1.145	1.143	1.146
Pub_year	.001	.000	3.261	1	.071	1.001	1.000	1.002
Constant	-6.099	.846	51.975	1	.000	.002		

**Fig 4:**
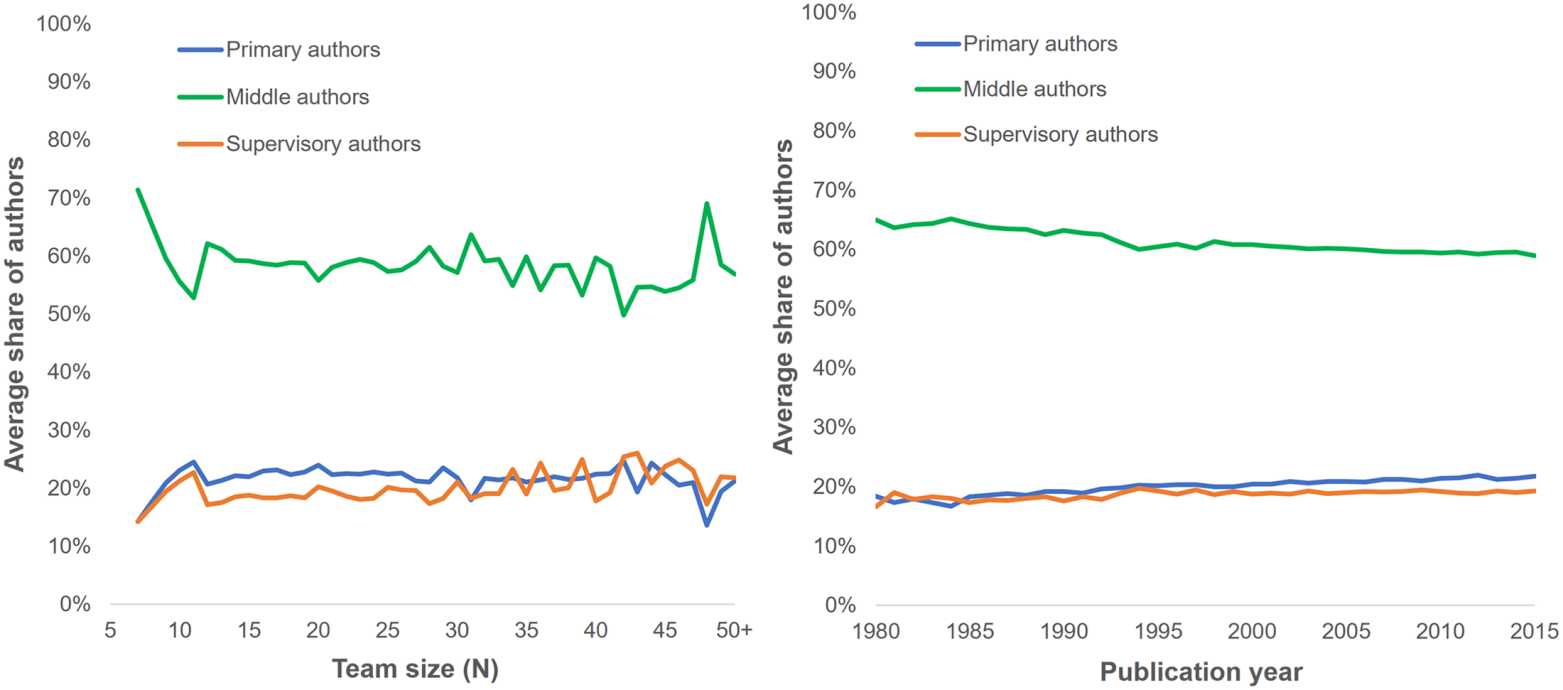
Average share of primary, middle and supervisory authors as a function of team size (left) and publication year (right).

Having established the high prevalence of bylines containing alphabetically ordered middle authors, we proceeded to analyze the team composition of articles where such sequences are found. This limited our analysis to the 74,555 articles containing a single alphabetical sequence for which *P*_*i*_ ≥ 0.95. Fig 4 displays the average proportion of primary, middle and supervisory author as a function of team size (left) and publication year (right). The results suggest that, independently of the team’s size, more than half the authors are middle authors. However, this proportion decreases slightly as team size increases. The other team members are distributed almost equally between primary and supervisory authors, the former being on average slightly more numerous than the former. Overall, the average team is composed of 20.9% (*SD* = .117) primary authors, 60.1% (*SD* = .141) middle authors, and 19.0% (*SD* = .119) supervisory authors.

The right panel of Fig 4 shows a slight decrease of the average proportion of middle authors in research teams, from 65.0% in 1980 to 59.0% in 2015. To disentangle the confounding effects of time and team size on the average proportion of middle authors, we performed a multiple regression to predict the share of middle authors using team size and publication year. The model shows a low negative effect of both independent variables, with F(2, 105,530) = 2.58x10e3, p <.001, adj. R2 = .047. Regression coefficients and standard errors are shown in Table 3.

**Table 3:** Coefficients of the multiple regression to predict the share of middle authors from the team size and the publication year.

	*B*	*SE*_*B*_	*β*	*P*
**Intercept**	4.458	.125		
**Team size (N)**	-.003	.000	-.185	.000
**Pub_year**	-.002	.000	-.093	.000

note: *B* = Unstandardized regression coefficient; *SE*_*B*_ = Standard error of the coefficient; *β* = Standardized coefficient.

**Fig 5:**
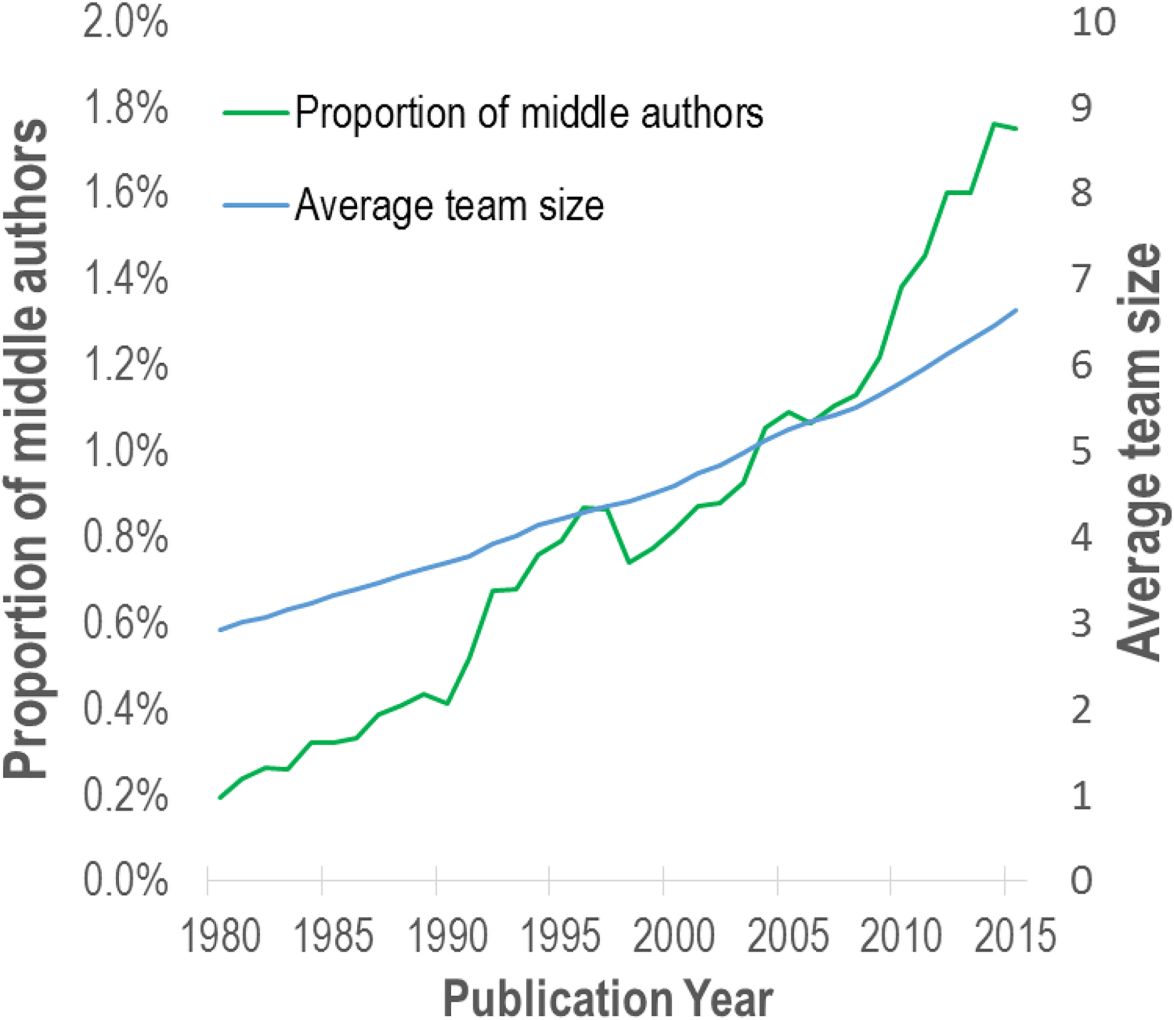
Evolution of the overall relative contribution of alphabetically ordered middle authors to biomedical research.

In this last part of our analysis, we look at the evolution over time of the overall contribution of middle authors to the biomedical literature. More specifically, we aim to determine whether the relative number of middle authors has been increasing at a lower, similar or higher rate than the average team size. For each year, we estimate the middle authors’ contribution to biomedical research by dividing the sum of all *r* for which *P*_*i*_ ≥ 0.95 by the sum of team size *N* for all biomedical articles in the WoS for that year (i.e., including those that do not contain an alphabetical sequence of authors). Fig 5 shows a ninefold increase of the contribution of alphabetically ordered middle authors to the biomedical literature over the 1980–2015 period. While they accounted for only 0.2% of all authors in 1980, they represented nearly 1.8% of authors in 2015. In comparison, the average team size has only doubled over the same period, going from 2.9 authors in 1980 to 6.6 authors in 2015. This suggests that over the last 35 years, middle authors have been playing an increasingly large part in the production of knowledge in the biomedical field.

One should note that while Fig 5 allows to draw the conclusion that the share of alphabetically ordered middle authors has been increasing, it does not provide an estimation of the total share of middle authors that are not listed in an alphabetical sequence since these middle authors are not identified with our data and methods.

## Discussion

On the byline of scholarly articles, the list of authors is the product of a combination of several factors. The nature, scope and complexity of a research project determine the amount of work, the various tasks and the array of knowledge and skills required. These may often be determinant factors in establishing the size of the research team and the division of labor among its members. Furthermore, the naming and ordering of authors, which we use to assess the relative contribution of team members, is the product of a more or less normalized social process. While strong (but implicit) disciplinary norms serve to guide authorship, other factors come to bear such as the existing relationships between collaborators and their position in the institutional hierarchy. We here discuss how these factors may explain our results.

In the first part of our analysis, we show a clear relation between the size of teams and the prevalence of alphabetically ordered middle authors. This can be explained by two different factors. Firstly, all other things being equal, a larger team will lead to a greater division of the work simply because the same amount of work can be divided among more team members. Uneven distribution of tasks will in turn determine author order, and allow for a distinction between primary, middle and supervisory authors. It is thus logical that the number of middle authors will increase as teams increase in size. Secondly, since it is more difficult to order very large number of authors based on their contributions, especially when contributions are small and diverse, it makes sense for the use of partial alphabetical order to increase with team size. Results also show that, for papers with 7 authors or more for which middle authors are listed in alphabetical order, these authors constitute the largest proportion of research teams (See Fig 4). This supports the idea that work is unevenly distributed among team members: a few primary authors lead the experimental work, others have a supervisory role, and the rest of the authors share smaller and/or more technical parts of the work.

However, a striking feature of the share of authors that fall in each of these three categories is their invariance as a function of team size. In other words, the increase in the number of authors per paper is not solely due to an increase in the number of middle authors but also to an equivalent proportional increase of primary and supervisory authors. This supports the hypothesis that larger teams might require a more diverse set of skills and roles including many primary authors and many supervisory authors.

Aside from considerations relating to the amount, complexity and division of the work, the large number of middle authors, especially in lengthy author lists, raises questions relating to authorship practices and criteria. It is possible, for instance, that the increasingly long bylines are not only reflecting an increase in collaboration, but also that small contributions are increasingly rewarded with authorship. Ordering middle authors alphabetically may reduce the incentive to keep the author list as short as possible because when authors are ordered according to their contribution or in full alphabetical order, adding a name on the byline reduces each one’s share of credit. Clearly distinguishing primary, middle and supervisory authors by using alphabetical order reduces this ‘loss of credit’ for primary and supervisory authors because they remain differentiated from the middle authors. Thus, listing middle authors in alphabetical order might increase the propensity to include more middle authors. Interestingly, this suggests that while the order of authors is determined by the type and amount of work, the method used for ordering names may also determine the type and amount of work required for an individual to be listed as an author. Another incentive for rewarding small contributions with authorship is the responsibility that is associated with authorship [17]. In a sense, naming all contributors as authors allows a more refined distribution of responsibility, where no author has to be accountable for the work of others.

The idea that partial alphabetical order would allow research teams to reward smaller (and perhaps non-substantial) contributions with authorship might seem somewhat at odds with the current authorship guidelines of the International Committee of Medical Journal Editors, which state that authorship is to be based on the following criteria:

- ‘Substantial contributions to the conception or design of the work; or the acquisition, analysis, or interpretation of data for the work; AND
- Drafting the work or revising it critically for important intellectual content; AND
- Final approval of the version to be published; AND
- Agreement to be accountable for all aspects of the work in ensuring that questions related to the accuracy or integrity of any part of the work are appropriately investigated and resolved.’ [30].

Previous studies have shown that honorary authors (defined as those who do not meet all ICMJE criteria) are present in about one fifth of biomedical papers [31,32]. It seems plausible that if partial alphabetical ordering of middle authors incentivizes the inclusion of more authors on papers, this may logically result in an increase in the prevalence of honorary authors. Such an apparent lack of adherence to authorship guidelines could suggest that it is time to re-examine their applicability.

More investigations will be necessary in order to better grasp how the empirical observations provided in this paper result from other factors such as the increased complexity of research, the increased division of labor, and a reduced threshold for inclusion in the authors list. Furthermore, as journals increasingly require that authors provide details about their contributions, including this information in further studies could also help better understand the relation between team size, individual contribution, and the use of partial alphabetical order.

Further research could also incorporate data on the authors’ affiliations to see how the number of departments or institutions involved in a project may relate to the division of labor, the use of partial alphabetical order, and the share of primary, middle and supervisory authors. We could hypothesize that research involving multiple institutions is more likely to bring together researchers with different expertise that will each play a major role (e.g. primary or supervisory). This could partly explain that increase in supervisory and primary authors observed in the present study.

## Conclusion

In this research, we demonstrated that the listing of middle authors alphabetically is a practice used frequently in biomedical research, especially in articles with a large number of authors. We also showed that when middle authors are identified alphabetically, they represent on average more than half of the research team. This indicates that the author inflation might be due in part to increased division of labor which may be associated to the increased size and complexity of research. This is reflected in the fact that the share of total authorships attributed to alphabetically ordered middle authors has increased more than average team size over the last 35 years. As discussed above, these results provide insights not only on collaboration and division of labor in biomedical research, but also on authorship practices.

The increase in teams’ size has raised issues that have been widely discussed, including the lack of transparency of authors’ contributions and the difficulties of assigning responsibility for the work as a whole or for its different parts. In addressing some of these issues, Baerlocher and colleagues [23] proposed that authors be designated as either primary, supervisory or contributing (middle) authors. We believe that such a system would be effective mainly in large teams as it would provide a normative framework that is more transparent and also better suited to reflect collaboration and division of labor in biomedical research. It would also provide recognition to individuals who make smaller contributions as contributing authors; this inclusive approach is more representative of various contributions than the current ‘all or nothing’ model that may exclude some contributors from the byline.

However, effective implementation of this model would require its acceptance by the scientific community and its adoption in research evaluation processes. Most of the currently used bibliometric indicators (e.g. the H-index) do not take into account one’s position on the byline. Consequently, being middle author may be paradoxically more rewarding, from a cost-benefit perspective, than being a primary or supervisory author. Inversely, indicators and evaluation processes that only put emphasis on the first position might create disputes and hinder collaboration and division of labor.

## Supporting information

S1 File Calculating the probability of chance and intentional alphabetical order. (PDF)

## References

[1] Lee S, Bozeman B. The Impact of Research Collaboration on Scientific Productivity. Soc Stud Sci. Sage Publications; 2005;35: 673–702. doi: 10.1177/0306312705052359

[2] de Beaver DB, Rosen R. Studies in scientific collaboration. Scientometrics. 1979;1: 133–149. doi: 10.1007/BF02016966

[3] Wuchty S, Jones BBF, Uzzi B. The increasing dominance of teams in production of knowledge. Science (80-). 2007;316: 1036–1039. doi: 10.1126/science.1136099 17431139 10.1126/science.1136099

[4] Larivière V, Gingras Y, Sugimoto CR, Tsou A. Team size matters: Collaboration and scientific impact since 1900. J Assoc Inf Sci Technol. 2015;66: 1323–1332. doi: 10.1002/asi.23266

[5] Jones BF, Wuchty S, Uzzi B. Multi-University Research Teams: Shifting Impact, Geography, and Stratification in Science. Science (80-). 2008;322: 1259–1262. doi: 10.1126/science.1158357 18845711 10.1126/science.1158357

[6] Adams JD, Black GC, Clemmons JR, Stephan PE. Scientific teams and institutional collaborations: Evidence from U.S. universities, 1981–1999. Res Policy. 2005;34: 259–285. doi: 10.1016/j.respol.2005.01.014

[7] Defazio D, Lockett A, Wright M. Funding incentives, collaborative dynamics and scientific productivity: Evidence from the EU framework program. Res Policy. 2009;38: 293–305. doi: 10.1016/j.respol.2008.11.008

[8] Katz JS, Martin BR. What is research collaboration? Res Policy. 1997;26: 1–18. doi: 10.1016/S0048-7333(96)00917-1

[9] Bozeman B, Corley E. Scientists’ collaboration strategies: implications for scientific and technical human capital. Res Policy. 2004;33: 599–616. doi: 10.1016/j.respol.2004.01.008

[10] Smith D, Katz JS. Collaborative Approaches to Research [Internet]. A Report to the Higher Education Funding Council for England. 2000. http://users.sussex.ac.uk/~sylvank/pubs/collc.pdf

[11] Cronin B. Hyperauthorship: A postmodern perversion or evidence of a structural shift in scholarly communication practices? J Am Soc Inf Sci Technol. 2001;52: 558–569. doi: 10.1002/asi.1097

[12] Maienschein J. Why collaborate? J Hist Biol. Springer; 1993;26: 167–183. doi: 10.1007/BF01061964

[13] Smith CG. Scientific Performance and the Composition of Research Teams. Adm Sci Q. 1971;16: 486 doi: 10.2307/2391768

[14] Larivière V, Desrochers N, Macaluso B, Mongeon P, Paul-Hus A, Sugimoto CR. Contributorship and division of labor in knowledge production. Soc Stud Sci. 2016;46: 417–435. doi: 10.1177/0306312716650046 10.1177/0306312716650046 28948891

[15] Rennie D, Yank V. If authors became contributors, everyone would gain, especially the reader. Am J Public Health. American Public Health Association; 1998;88: 828–830. doi: 10.2105/AJPH.88.5.828 10.2105/ajph.88.5.828 PMC1508948 9585760

[16] Cronin B. Rates of return to citation. J Doc. 1996;52: 188–197. doi: 10.1108/eb026967

[17] Birnholtz JP. What does it mean to be an author? The intersection of credit, contribution, and collaboration in science. J Am Soc Inf Sci Technol. Hoboken; 2006;57: 1758–1770. doi: 10.1002/asi.20380

[18] Merton RK. Priorities in scientific discovery: A chapter in the sociology of science. Am Sociol Rev. 1957;22: 635 doi: 10.2307/2089193

[19] Cetina KK. Epistemic cultures: Forms of reason in science. Hist Polit Econ. 1991;23: 105–122. doi: 10.1215/00182702-23-1-105

[20] Pontille D. La signature scientifique: une sociologie pragmatique de l’attribution. Paris: CNRS; 2004.

[21] Ivaniš A, Hren D, Sambunjak D, Marušić M, Marušić A. Quantification of Authors’ Contributions and Eligibility for Authorship: Randomized Study in a General Medical Journal. J Gen Intern Med. Springer; 2008;23: 1303–1310. doi: 10.1007/s11606-008-0599-8 18521691 10.1007/s11606-008-0599-8 PMC2518038

[22] Ivaniš A, Hren D, Marušić M, Marušić A. Less Work, Less Respect: Authors’ Perceived Importance of Research Contributions and Their Declared Contributions to Research Articles. Ross JS, editor. PLoS One. 2011;6: e20206 doi: 10.1371/journal.pone.0020206 21713036 10.1371/journal.pone.0020206 PMC3119662

[23] Baerlocher MO, Newton M, Gautam T, Tomlinson G, Detsky AS. The Meaning of Author Order in Medical Research. J Investig Med. LWW; 2007;55: 174–180. doi: 10.2310/6650.2007.06044 17651671 10.2310/6650.2007.06044

[24] Mongeon P, Larivière V. Costly collaborations: The impact of scientific fraud on co-authors’ careers. J Assoc Inf Sci Technol. 2016;67: 535–542. doi: 10.1002/asi.23421

[25] Boyack KW, Klavans R, Sorensen AA, Ioannidis JPA. A list of highly influential biomedical researchers, 1996–2011. Eur J Clin Invest. 2013;43: 1339–1365. doi: 10.1111/eci.12171 24134636 10.1111/eci.12171

[26] Warner ET, Carapinha R, Weber GM, Hill E V, Reede JY. Faculty Promotion and Attrition: The Importance of Coauthor Network Reach at an Academic Medical Center. J Gen Intern Med. 2016;31: 60–67. doi: 10.1007/s11606-015-3463-7 26173540 10.1007/s11606-015-3463-7 PMC4700018

[27] Pontille D. Qu’est-ce qu’un auteur scientifique. Sci la Société. 2006;67: 77–93.

[28] Zuckerman HA. Patterns of name ordering among authors of scientific papers: A study of social symbolism and its ambiguity. Am J Sociol. The University of Chicago Press; 1968;74: 276–291.

[29] Waltman L. An empirical analysis of the use of alphabetical authorship in scientific publishing. J Informetr. 2012;6: 700–711. doi: 10.1016/j.joi.2012.07.008

[30] International Committee of Medical Journal Editors. Recommendations for the conduct, reporting, editing, and publication of scholarly work in medical journals [Internet]. 2015 [cited 28 Aug 2016]. http://www.icmje.org/icmje-recommendations.pdf 25558501

[31] Flanagin A, Carey LA, Fontanarosa PB, Phillips SG, Pace BP, Lundberg GD, et al Prevalence of articles with honorary authors and ghost authors in peer-reviewed medical journals. JAMA. Am Med Assoc; 1998;280: 222 doi: 10.1001/jama.280.3.222 10.1001/jama.280.3.222 9676661

[32] Wislar JS, Flanagin A, Fontanarosa PB, DeAngelis CD. Honorary and ghost authorship in high impact biomedical journals: a cross sectional survey. BMJ. BMJ Group; 2011;343: d6128–d6128. doi: 10.1136/bmj.d6128 22028479 10.1136/bmj.d6128 PMC3202014

