# Supplementary material for: The rise of the middle author: Investigating collaboration and division of labor in biomedical research using partial alphabetical authorship
Source: PLoS One. 2017 Sep 14;12(9):e0184601. doi: 10.1371/journal.pone.0184601 (PMC5599011; doi:10.1371/journal.pone.0184601)
Supplement: S1 File — (PDF) [file pone.0184601.s001.pdf]

# Calculating the probability of chance and intentional alphabetical order

Let $n \geq 1$ be an integer and let $S_n$ denote the set of permutations of $\{1,\ldots,n\}$. Let us write

$$\sigma = [a_1 \; a_1 \; \ldots a_n]$$

for the permutation with $\sigma(i) = a_i$. For $1 \leq r <= n$, we shall say that σ has an increasing subsequence of length *r* if there exists *r* consecutive terms in increasing order:

$$a_j < a_{j+1} < \cdots < a_{j+r-1}.$$

Let us denote $S_n(r) \subseteq S_n$ the subset consisting of those permutations with an increasing subset of length *r*. For instance, we have

$$S_4(3) = \{[1\ 2\ 3\ 4], [4\ 2\ 1\ 3], [3\ 1\ 2\ 4], [2\ 1\ 3\ 4], [2\ 3\ 4\ 1], [1\ 2\ 4\ 3]\}.$$

Let $s_n(r)$ denote the number of elements of $S_n(r)$, so for instance, we have $s_4(3) = 7$. We have $s_n(n) = 1$ since there can only be one entirely increasing permutation. On the other hand, we have $s_n(1) = !\,n$ because every permutation trivially contains a 1-term increasing subsequence.

Define $b_n(r) = n! - s_n(r)$, which is the number of permutations of *n* authors which do *not* contain an alphabetically ordered subsequence of *r* authors. One can show by a combinatorial argument (See Elizalde and Noy, 2003) that the exponential generating function

$$B_r(X) \stackrel{\text{def}}{=} \sum_{n \geq 0} b_n(r) \frac{X^n}{n!}$$

can be written as

$$B_r(X) = \left( \sum_{n \geq 0} \frac{X^{nr}}{(nr)!} - \frac{X^{nr+1}}{(nr+1)!} \right)^{-1}$$

Using this formula, we computed $b_n(r)$ with the computer algebra system SAGE[1]. The Probability of intentional alphabetical order $P_i$ is thus obtained with the following formula:

$$P_i = \frac{b_n(r)}{n!}$$

Inversely, the probability of chance alphabetical order $P_c$ is obtained with the following formula:

$$P_i = \frac{n! - b_n(r)}{n!}$$

**Reference**


Elizalde, S., Noy, M. (2003). Consecutive patterns in permutations. Advances in Applied Mathematics, *30*(1-2), 110-125.


[1] Sage Mathematics Software (Version 7.1), The Sage Developers, http://www.sagemath.org.
